# Adaptive effort investment in cognitive and physical tasks: a neurocomputational model

**DOI:** 10.3389/fnbeh.2015.00057

**Published:** 2015-03-09

**Authors:** Tom Verguts, Eliana Vassena, Massimo Silvetti

**Affiliations:** Department of Experimental Psychology, Ghent UniversityGhent, Belgium

**Keywords:** cognitive effort, cognitive control, physical effort, computational model

## Abstract

Despite its importance in everyday life, the computational nature of effort investment remains poorly understood. We propose an effort model obtained from optimality considerations, and a neurocomputational approximation to the optimal model. Both are couched in the framework of reinforcement learning. It is shown that choosing when or when not to exert effort can be adaptively learned, depending on rewards, costs, and task difficulty. In the neurocomputational model, the limbic loop comprising anterior cingulate cortex (ACC) and ventral striatum in the basal ganglia allocates effort to cortical stimulus-action pathways whenever this is valuable. We demonstrate that the model approximates optimality. Next, we consider two hallmark effects from the cognitive control literature, namely proportion congruency and sequential congruency effects. It is shown that the model exerts both proactive and reactive cognitive control. Then, we simulate two physical effort tasks. In line with empirical work, impairing the model's dopaminergic pathway leads to apathetic behavior. Thus, we conceptually unify the exertion of cognitive and physical effort, studied across a variety of literatures (e.g., motivation and cognitive control) and animal species.

## Introduction

Adaptive choice requires deciding how much effort to invest. Do we ride our bike extra fast to reach the supermarket before it closes? In the supermarket, do we mentally calculate price differences between brands up to the eurocent? Several studies investigated the correlates of effort exertion. Effort is exerted for difficult and highly rewarding tasks (Pessiglione et al., 2007; Krebs et al., 2012; Salamone and Correa, 2012; Vassena et al., 2014). Effort investment varies across individuals (Boksem and Tops, 2008; Treadway et al., 2012b), and is impaired in clinical conditions like chronic fatigue syndrome, ADHD, and depression (Volkow et al., 2011; Treadway et al., 2012a). Effort can be pharmacologically manipulated (Bardgett et al., 2009; Salamone and Correa, 2012). However, a mechanistic understanding of how organisms learn to exert effort is lacking. To address this issue, we frame effort allocation as a reinforcement learning (RL) problem (Sutton and Barto, 1998). In RL, an action is chosen that maximizes some value (utility) function. We consider optimization of a value function combining reward and effort cost (Rigoux and Guigon, 2012). Effort is indeed a cost dimension that subjects try to minimize (Kool et al., 2010). We consider deciding to apply effort as an action to be chosen. Indeed, although effort is no overt response, it has effects on the environment, so RL principles can be applied.

We start from an optimality consideration of effort, specifying what a neurocomputational model might approximate. Next, we propose a neural substrate for effort. The basal ganglia (BG)-thalamocortical circuit through the anterior cingulate cortex (ACC) and BG is ideally suited for this purpose (Alexander et al., 1986). This *limbic loop* originates in ACC, and subsequently projects to ventral striatum (VS), ventral pallidum (VP), and thalamus (GABA inhibitory pathways). The thalamus projects to ACC, thus closing the loop. The brainstem dopaminergic ventral tegmental area (VTA) projects into this limbic loop, in particular to ACC and VS (Pierce and Kumaresan, 2006). Classically, the limbic loop is considered a gateway between emotion and action (Mogenson et al., 1980). More specifically, it processes effort. For example, dopaminergic depletion of VS impairs effort (Salamone et al., 1994; Denk et al., 2005; Bardgett et al., 2009) and dopaminergic stimulation of VS increases effort (Bardgett et al., 2009; Wardle et al., 2011). Excitotoxic ACC lesions also impair effort (Walton et al., 2002, 2009). In humans, the absence of effort initiation is a hallmark of akinetic mutism, which is also associated with ACC lesions (Devinsky et al., 1995). Stimulation of ACC induces the “will to persevere,” a subjective feeling of increased motivation (Parvizi et al., 2013). VS is activated in anticipation of high effort (Boehler et al., 2011). Besides effort, the limbic loop also processes reward (Kable and Glimcher, 2007; Matsumoto et al., 2007; Pessiglione et al., 2007; Croxson et al., 2009).

To understand the limbic loop and its role in effort processing, we seek inspiration from data and theory concerning another cortico-striato-pallido-thalamo-cortical pathway, the motor loop (Alexander et al., 1986). The motor loop receives dopaminergic input from VTA and substantia nigra (SN). Earlier computational models (Frank, 2005; Ashby et al., 2007) describe how this motor loop trains cortical pathways. We propose that the limbic loop also modulates cortical pathways, not by training them but by effort modulation. In this way, reward and cost feedback is used both for learning a stimulus-action mapping (motor loop) and for choosing when and whether to invest effort (limbic loop).

The optimality principle describes *why* a cognitive system should allocate effort for what tasks; the neurocomputational model states *how*. We next consider *what* types of effort exist. Cognitive effort is often studied using congruency tasks like the Stroop, Simon, or flanker tasks. In the Stroop task, subjects see a colored word (e.g., RED) and must inhibit reading the word and instead name the ink color. The congruency effect entails that performance is better and easier when word and ink color point to the same response (e.g., congruent stimulus RED is easier than incongruent stimulus RED; Egner, 2008). In addition, we consider two hallmark findings, proportion congruency and sequential congruency effects. The proportion congruency effect means that the congruency effect is smaller in blocks with more incongruent stimuli. A common interpretation is that subjects prepare for incongruent trials in difficult blocks, exerting proactive control (Cheesman and Merikle, 1986; Bugg and Chanani, 2011; Braver, 2012). The sequential congruency effect entails that the congruency effect is smaller after an incongruent trial. This is often interpreted as an instance of reactive control (Ansorge et al., 2011; Braver, 2012). After a difficult trial, subjects temporarily exert extra effort.

Physical effort is commonly studied in rodents (Salamone and Correa, 2012; but see Meyniel et al., 2014). We also consider two hallmark findings from this literature, effort discounting and hyperbolic responding in variable-interval schedules. Effort discounting entails that animals are willing to work for reward as long as it is sufficiently large relative to the required effort. However, animals with a dopaminergically depleted limbic loop refuse working and instead go for the easier but less rewarding option (Salamone et al., 1994; Walton et al., 2009, 2002). In the variable-interval reinforcement schedule (Catania and Reynolds, 1968), animals perform some action repetitively (e.g., lever pressing), and are reinforced with food on the first lever press after a variable (randomly selected) interval. Here also, balancing reward and effort costs is essential. This balance leads to hyperbolic responding: Response rate increases almost linearly as a function of reward rate at small reward rates, but plateaus when reward rate is increased further. Intuitively, at some point increased responding is no longer worth the extra (effort) cost.

## Optimality

We aim to determine how to optimally allocate effort as a function of a reward, cost, and task difficulty. Consider the utility function for a given task, as a function of effort:

(1) U(effort) =E(reward)−effort cost

where *E*(reward) expresses the expected reward. We assume reward is contingent on solving the task, and solving it occurs with some probability. This probability is a monotonically increasing function of effort; and a monotonically decreasing function of task difficulty. Further, we assume that effort cost is a linear function of effort level. Equation (1) thus becomes

(2) $$U(effort)\;=r\frac{effort}{difficulty+effort}-c\cdot effort$$

The parameters *r* and *c* linearly scale expected reward and effort cost, respectively. The cost parameter *c* may depend on various factors, including long-term fatigue (due to prolonged effort exertion), short-term fatigue (the time since the last effort expenditure, as in Simulation 5) or individual differences. Note that this utility function is inherently subjective (subject-dependent): Both effort and difficulty depend on the subject's capacities and prior exposure in the task at hand.

The effort that maximizes Equation (2) is the *optimal effort*, and it turns out to be

(3) $$optimal\;effort=\sqrt{\frac{difficulty\cdot r}{c}}-difficulty$$

**Figure 1:**
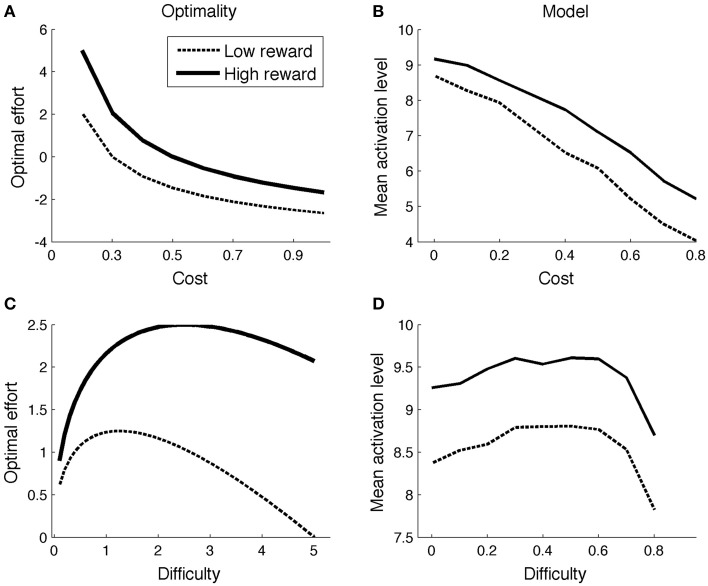
**Comparison of optimality perspective (A,C) and Simulation 1 (B,D)**.

With this result, one can determine the optimal effort level for various combinations of reward (*r*), cost (*c*), and task difficulty. Figure 1A illustrates the effect of reward and cost (*difficulty* = 5; low reward = 1; high reward = 2). The optimal effort level is a decreasing function of cost, and an increasing function of reward. Figure 1C shows optimal effort levels for different levels of task difficulty (*c* = 0.2; low reward = 1; high reward = 2). The shape of the curve is inverted-U: For very easy tasks, it is not worth putting in effort, as they will be solved correctly even without effort. On the other hand, for very difficult tasks, effort is not worth it either: They will not be finished successfully anyway.

## Neurocomputational model

**Figure 2:**
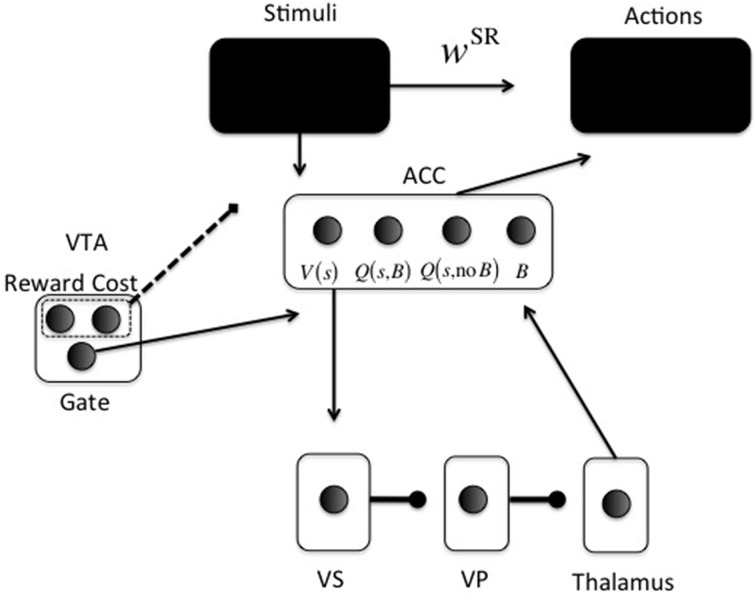
**Neurocomputational model**. Arrowheads are excitatory; circleheads are inhibitory; the dashed line represents reward and cost feedback (i.e., input for learning). The two black boxes are opened in different simulations. The ACC representation is shown for just one stimulus *s*: One unit for *V*(*s*), one for *Q*(*s*, Boost), one for *Q*(*s*, No Boost), and one for boosting the action layer (*B*).

In the neurocomputational model, one part implements the minutiae of the relevant task (upper part in Figure 2), which is different for each simulation. This is encoded by parameter *w*^SR^, which are neural network weights between stimulus and action representations. This mapping is assumed to be learned earlier, and via pathways like the motor loop. Specific mappings are reported for each simulation below.

The second part (lower part in Figure 2) is the focus of the current paper. It determines whether investing effort for the current configuration of reward, cost, and task difficulty, is worth it. For this purpose, cortical stimulus areas project to ACC. The ACC represents both stimulus value [*V*(*s*)] and values of actions for a given stimulus [*Q*(*s*, *a*)].

A recent debate centers on what effort is exactly; an energetic cost (Laughlin et al., 1998), an opportunity cost (Kurzban et al., 2013), or both (Niv et al., 2007; Dayan, 2012). Our implementation is compatible with either, and derives from earlier work on attentional processing. It consists of increasing signal-to-noise ratio (SNR) in cortical areas (McClure et al., 2005; Cohen et al., 2007), called *boosting* for short. For simplicity, boosting is not continuous, but instead Boost and No Boost are two discrete actions. These actions will be called *options* to avoid confusion with cortical actions (Figure 2). ACC learns the (*Q*-)value of each option for specific stimuli or contexts. A key point of the model is that effort is modularized, in the sense that effort and its associated effort cost are processed separately from the task to be solved. Thus, we implement a “divide and conquer” strategy in which different anatomical regions learn different regularities. Effort is also processed separately from other value dimensions and indeed other cost dimensions, such as delay cost. Even though delay cost is not explicitly implemented here, it is clear that effort and delay costs would be processed separately in the current modeling framework, simply because they have different functional consequences. An organism should respond differently to an effort cost (e.g., climbing) than to a delay cost (e.g., waiting). This principle is compatible with a host of data showing explicit dissociations between the two dimensions of cost (Rudebeck et al., 2006; Floresco et al., 2008; Prévost et al., 2010).

In the model, ACC projects directly to the VS (nucleus accumbens) (Heimer et al., 1997; Basar et al., 2010). From VS, an inhibitory pathway projects to ventral pallidum (VP), which inhibits the thalamus (Alexander and Crutcher, 1990). This is called the direct pathway in the BG; the indirect pathway is not currently implemented.

When state and action transition probabilities are known, one can find the values of a policy by solving a set of Bellman equations (Sutton and Barto, 1998). However, these probabilities are typically unknown. Besides, even if they were known, an organism busy interacting with its world does not straightforwardly solve the equations. Fortunately, an alternative and more practical method exists, called Monte-Carlo RL. It involves sampling the environment to update value estimates of one's policy. In theory, one alternates between estimating the value of a complete policy with updating this policy based on the value estimates. In real life, however, this is typically not feasible. As an alternative, one can mix value estimation and policy updating by estimating (*Q*-)values and updating the policy at every trial, which is called generalized policy iteration (GPI). GPI leads to an optimal policy (Sutton and Barto, 1998), and we implement it here. An option *a* ϵ {Boost, No Boost} is chosen in ACC using a softmax rule:

(4) $$\mathrm{Pr}(a)\;=\;\frac{\exp\left(\gamma Q_{n}^{\pi }(s,a)\right)}{\sum_{a^{\prime }}\exp\left(\gamma Q_{n}^{\pi }(s,a^{\prime })\right)}$$

Thus, the continuous variable *Q*^π^_*n*_ (*s, a*) is transformed into a binary activation. A lower gain parameter γ means lower SNR and more exploration in choosing an option. When the Boost option is chosen, the boost value unit [*Q*(*s*, *B*) in Figure 2] in ACC is activated (*x*_Boost_ = 1). Otherwise, the no-boost value unit [*Q*(*s*, no *B*] in Figure 2) in ACC is activated (*x*_NoBoost_ = 1). The boost option is thus chosen by ACC. It subsequently determines VS activation in the following way (using fixed weights from ACC to VS): VS = 10*x*_Boost_ + *x*_NoBoost_ This activation is linearly passed on from VS to VP and to thalamus, and back to ACC to activate the boosting unit in ACC (*B* in Figure 2) with value *ACC*_Boost_. The gate in VTA (Figure 2) is made explicit when we explain the dopaminergically lesioned model (Simulation 4). Finally, this activation modulates the choice for an appropriate action (“Actions” box in Figure 2). Action *k* is also chosen via a softmax rule:

(5) $$\mathrm{Pr}(k)\;=\frac{\exp\left(ACC_{Boost}{\sum_{i}w}_{ik}^{\text{SR}}x_{i}\right)}{\sum_{k^{\prime }}\exp\left(ACC_{Boost}\sum_{i}w_{ik^{\prime }}^{\text{SR}}x_{i}\right)}$$

in which **x** is a vector with *x_i_* = 1 if stimulus *i* is presented and zero otherwise.

Note that ACC activation does not choose the action *k*, but serves as a gain parameter that determines SNR in the pathway implementing the task.

Learning to exert effort is supported by reward and cost feedback from VTA dopaminergic input into ACC (Takahata and Moghaddam, 2000; Düzel et al., 2009). VTA responds to both reward (Schultz et al., 1997) and cost (Matsumoto and Hikosaka, 2009), including effort cost (Krebs et al., 2012). Dopaminergic projections train the limbic loop (Schultz et al., 1997), like they train the motor loop (Frank, 2005; Ashby et al., 2007). Option values are updated as:

(6) $$Q_{n}^{\pi }(s,a)=Q_{n-1}^{\pi }(s,a)\;+\;\alpha \;\left(R-c\cdot x_{Cost}-Q_{n-1}^{\pi }(s,a)\right)$$

Here, *Q*^π^_*n*_ (*s, a*) is the value (averaged and cost-discounted reward) of choosing option *a* when confronted with stimulus *s*, if one chooses policy π afterwards. Reward and (effort) cost are detected in VTA; the factor *R* – *c.x*_cost_ implements the value (reward—effort cost) that was obtained (dotted line in Figure 2). For simplicity cost is zero (*x*_cost_ = 0) in case of no boosting; effort cost scales linearly with parameter *c* (*x*_cost_ = 1;) in case of boosting.

## Simulation 1: optimizing value by adaptive effort allocation

### Methods

**Figure 3:**
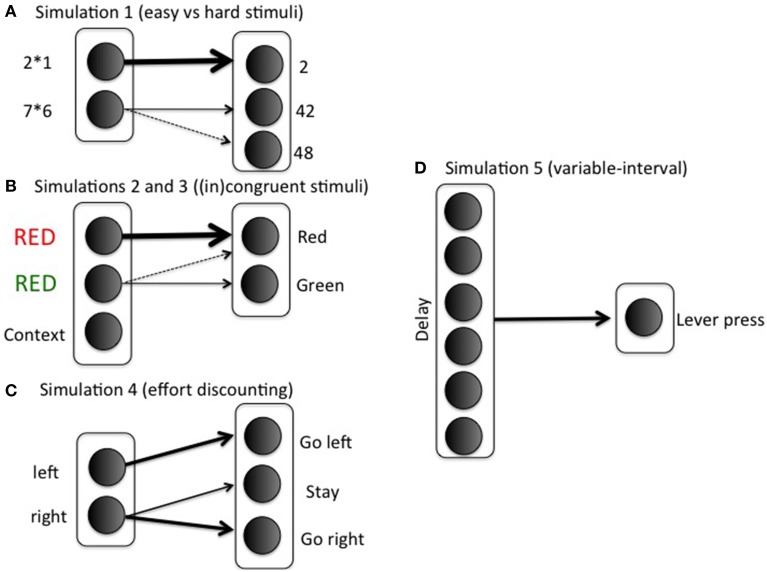
**Simulation-specific stimulus-action mappings**.

We investigate the model's effort investment for different combinations of reward, cost, and task difficulty. The task-specific stimulus-response matrix w^SR^ [used in Equation (5)] determines difficulty. For example, if there is one easy (e.g., a simple arithmetic problem, 2 × 1), and one difficult stimulus (e.g., a more difficult problem, 7 × 6), the weight matrix could be

(7) $$w^{\text{SR}}=\left(\begin{array}{ccc} 10 & 0 & 0\\ 0 & 1 & 0.8 \end{array}\right)$$

with rows indexing stimuli (*i*; 2 × 1, 7 × 6) and columns indexing response options (*k*; 2, 42, 48). The first row corresponds to the easy stimulus; in general, if *w*^*SR*^_12_ = *w*^*SR*^_13_ = 0, then any value *w*^*SR*^_11_ > ln(2)/β will lead to probability of success >1/2, where β is the gain parameter (implemented as ACC_*boost*_ in Equation 5). This lower bound ln(2)/ β is difficult to determine exactly, because the gain parameter is optimized using reinforcement learning during the task (Equation 5). For this reason, we set *w*^*SR*^_11_ to a sufficiently high value, so probability of success is always high for easy stimuli. The 0.8 in row 2 indicates a possible confusion between the actions “42” and “48” (e.g., Campbell and Graham, 1985). Note that we represent just a small subset of the mental arithmetic network, and with only limited realism. The modeling focus is on effort rather than arithmetic, so we keep the task settings as minimal as possible. Figure 3A illustrates the specific mapping.

In Simulation 1a, we varied the effort cost *c* for boosting from 0 to 0.8 (cost of not boosting is zero). We used two stimuli, one low reward (reward = 1.5 for correct response), one high reward (reward = 2 for a correct response). Both stimuli were difficult (row 2 of Equation 7). Learning rate (α) and gain parameter (γ) were set at 0.5 and 3, respectively. We ran 150 training trials, followed by 50 test trials in which data were recorded. Results are averaged across 1000 replications of this procedure.

In Simulation 1b, we manipulated difficulty. In particular we changed the 0.8 in Equation (7) to the parameter δ, which indexed task difficulty. A value δ = 0 means a relatively easy task; δ = 1 means an impossibly difficult task. The cost (*c*) for effort is arbitrarily fixed at 0.2 for boosting.

### Results and discussion

#### Simulation 1a

Figure 1B displays mean ACC activation (here and elsewhere, measured as *ACC*_Boost_) for different levels of reward and cost. Because the scales of the optimal (left column) and neurocomputational (right column) models are different, and because difficulty is scaled in a different way, a quantitative comparison between the two models is uninformative. However, both show the same qualitative trends (more activation for high reward and low cost). The effect of more activation for high reward than for low reward is consistent with empirical data (Knutson et al., 2001, 2005; Krebs et al., 2012). The effect of cost stands out as an empirical prediction.

#### Simulation 1b

Figure 1D shows the model's allocated effort level as a function of task difficulty. Intuitively, when a task is very difficult, it's not even worth trying (to boost). When a task is very easy, no effort is needed, because the task will be solved correctly anyway. Hence, the allocated effort level exhibits an inverted-U shape. Part of the pattern has been empirically verified. When task difficulty is low-to-moderate (i.e., accuracy levels much higher than chance level), ACC is more active for (moderately) difficult than for easy tasks (Boehler et al., 2011; Vassena et al., 2014). The neurocomputational model does not completely conform to the optimal model, however. In the optimal model, effort is an increasing function of difficulty for a larger range of parameters for high reward than for low reward (Figure 1C). This does not appear to be true in the neurocomputational model (Figure 1D). Future studies should determine more precisely the relationships between the optimal model, the neurocomputational model, and corresponding empirical results.

## Simulation 2: blockwise proportion congruency effect

### Methods

As noted above, the proportion congruency (PC) effect means that task blocks with more incongruent (MI) trials exhibit a smaller congruency effect than blocks with more congruent (MC) trials (Logan and Zbrodoff, 1979; Tzelgov et al., 1992; Bugg et al., 2011). It has been interpreted as an instance of proactive control, meaning that participants exert more control (effort) in blocks with mostly incongruent trials than in blocks with mostly congruent trials (Bugg et al., 2011; Braver, 2012). Some authors have argued that the effect derives from the relative stimulus frequencies (Schmidt and Besner, 2008). However, the PC effect generalizes to stimuli with matched frequencies in the same block (Bugg and Chanani, 2011; Bugg and Crump, 2012), so this cannot be its only origin.

We used the S-R matrix

(8) $$w^{\text{SR}}=\left(\begin{array}{cc} 10 & 0\\ \delta & 1 \end{array}\right)$$

with δ again indexing difficulty, and easy and difficult stimuli corresponding to congruent and incongruent trials, respectively. Columns 1 and 2 correspond to the correct responses for stimuli 1 and 2, respectively. See Figure 3B for an illustration of the mapping in the context of a Stroop task. As in the arithmetic example, this implementation is not intended to imply much realism. Solving incongruent stimuli involves top-down control signals which bias bottom-up processing pathways toward the less dominant but correct response (Miller and Cohen, 2001). However, such top-down signals are not the focus of this paper. The modular model structure allows implementing any task in a convenient stimulus-action weight matrix to which the effort processing machinery can be hooked up. In such a weight matrix, the difference between correct response and incorrect response should be much smaller for an incongruent than for a congruent stimulus; however, the correct response should still be more likely than the incorrect response in both cases. Otherwise, accuracies would be below chance level, which is inconsistent with empirical data. To sum up, the stimulus-action weight matrix is a simple way of implementing bottom up/top down interactions with two main features: Congruent is easier than incongruent; but correct response is always more likely than incorrect. The Appendix presents a more formal argument that the current S-R matrix implements a combined “bottom-up” and “top-down” configuration.

We introduce a context unit that is always active in a task block (Figure 3B). It obeys the same learning laws as other stimuli (Equation 6). Boosting is determined by the Q-values for boosting vs. not boosting for this context unit. This means that, in the current simulation, only the block context (rather than the stimulus) determines whether to boost or not. This is of course a simplifying assumption: In general, we expect that both task and stimulus context determine whether to exert effort or not. However, given that the current simulation focused on task context, we opted for the simplest choice and implemented an effect of task context only. Easy and difficult stimuli (Equation 8) were randomly presented for 200 trials (80% easy stimuli in MC blocks; 20% easy stimuli in MI blocks). Hundred simulations were run and averaged.

To explore the model's parameter space, the parameters reward, cost, difficulty, and learning rate were systematically varied. As an approximation to this four-dimensional space, we plot model performance in two of its subspaces. In the first (two-dimensional) subspace, we vary the reward (*R* in Equation 6) of the difficult stimulus; the reward of the easy stimulus is 1. In this first subspace, we also vary the difficulty of the difficult stimulus (δ).

In the second subspace, we vary the learning rate (α in Equation 6) and we vary the cost parameter (*c* in Equation 6); here we set reward for the easy and difficult stimulus to 1 and 2 respectively, and δ = 0.8.

We plot accuracy for each point in the parameter space. In addition, we plot the congruency effect, that is, the mean difference in accuracy for congruent vs. incongruent stimuli. Finally, we plot the critical PC interaction (*X^MC^_c_* − *X^MC^_i_*) − (*X^MI^_c_* − *X^MI^_i_*) where *X^MC^_c_* and *X^MI^_c_* are the mean accuracies for congruent stimuli in MC and MI blocks, respectively (similar for incongruent stimuli, with subscript *i*). Positive values of this contrast indicate consistency of the model with the PC effect (smaller congruency effect in MI blocks relative to MC blocks; Tzelgov et al., 1992; Bugg and Chanani, 2011).

### Results and discussion

ACC is more active for incongruent relative to congruent stimuli (Van Veen and Carter, 2002; Kerns et al., 2004). The same is true in the model, for the same reason as in Simulation 1: In the low-to-moderate difficulty range where accuracies are well above chance level (Figures 1C,D), more effort is exerted for difficult than for easy stimuli.

**Figure 4:**
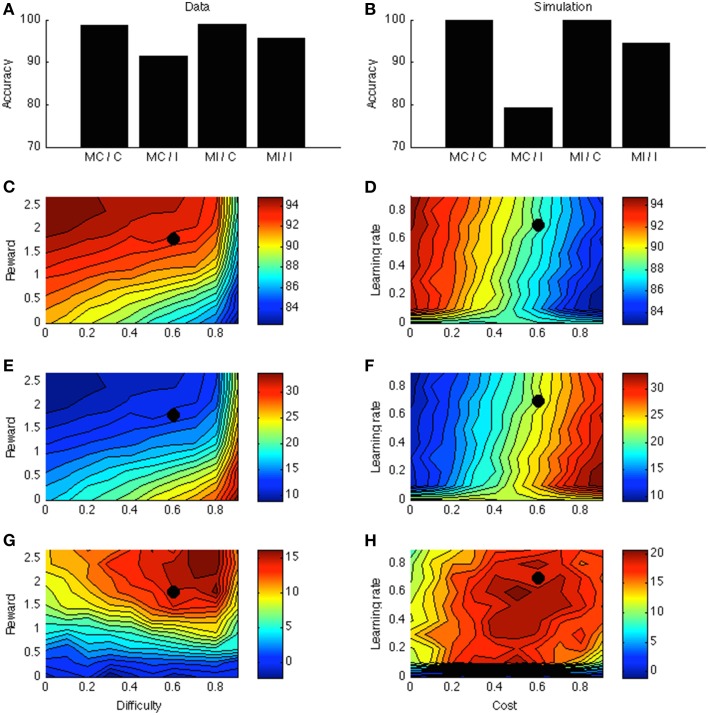
**(A)** Proportion congruency data from Bugg and Chanani. Labels C and I indicate congruent and incongruent, respectively. **(B)** Simulated PC effect. **(C)** Simulated accuracy across different levels of reward and task difficulty. **(D)** Simulated accuracy across different levels of learning rate and cost. **(E)** Simulated congruency effect across reward and difficulty. **(F)** Simulated congruency effect across learning rate and cost. **(G)** Simulated PC effect across reward and difficulty. **(H)** Simulated PC effect across learning rate and cost. Black dots in **(C–H)** indicate the parameter used to generate **(B)**.

Figure 4A displays the PC effect from a typical data set (Bugg and Chanani, 2011). Only accuracy data (rather than response times) are plotted because the current version of the model generates accuracy data only. Figure 4B shows the simulation results from one point in the parameter space; this point is indicated by a black dot in Figures 4C–H. Figure 4C shows that accuracy is higher for higher rewards. This is consistent with empirical literature (Krebs et al., 2010; Padmala and Pessoa, 2011). In the simulation (Figure 4E), the congruency effect is smaller when rewards are larger, again consistent with data (Padmala and Pessoa, 2011). Figure 4G shows that the PC effect is larger for bigger rewards. This remains a prediction for future empirical investigation.

Figures 4D,F,H shows that learning rate has little effect on accuracy, congruency, or PC effects. The only exception is when learning rate is very low, in which case the model cannot learn the block contingencies. Finally, cost has a strong (and opposite) effect on accuracy and congruency effect, but little on the PC effect. These predictions remain open for future investigation.

In general, reward, cost, difficulty, and learning rate have orderly influences on task performance. Context was here simply implemented as a unit active throughout the block, and for simplicity, only this unit was associated to the ACC. More generally, however, also the task stimuli or other contextual elements are likely to be associable to ACC effort neurons, consistent with a broad empirical literature (Crump et al., 2006; Crump and Milliken, 2009). For example, if more incongruent stimuli appear in the upper than in the lower location, stimuli at the upper location have a smaller congruency effect (even in stimuli with matched frequencies) (Crump and Milliken, 2009). Lehle and Huebner (2008) demonstrate a similar effect for (irrelevant) color rather than location cues. Such context effects can be easily implemented in the current model.

## Simulation 3: the sequential congruency effect

### Methods

Besides the proportion of congruent and incongruent trials in a block (Simulation 2), also congruency of the previous trial influences current-trial congruency. In particular, the congruency effect is smaller after an incongruent trial (Gratton et al., 1992), called the sequential congruency effect or Gratton effect (Notebaert and Verguts, 2008; Fischer et al., 2010). It is a major inspiration for theories of cognitive control and ACC (Botvinick et al., 2001; Egner and Hirsch, 2005; Verguts and Notebaert, 2008; Grinband et al., 2011). It is often interpreted as an instance of reactive control (Braver, 2012): Because of difficulty experienced on the previous trial, subjects invest more effort on the next. Low-level trial-to-trial priming effects (Mayr et al., 2003) do not account (completely) for the sequential congruency effect (Ullsperger et al., 2005; Notebaert and Verguts, 2007; Duthoo and Notebaert, 2012).

Model and parameter settings were the same as in Simulation 2 except that (as in empirical paradigms for sequential congruency) the proportion of congruent stimuli was 50%. Besides accuracy and congruency effect, we now plot the critical interaction contrast (*X^pC^_c_* − *X^pC^_i_*) − (*X^pI^_c_* − *X^pI^_i_*) where *X^pC^_c_* and *X^pI^_c_* are mean accuracies for congruent stimuli, if the previous trial was congruent vs. incongruent, respectively (similar for incongruent stimuli, subscript *i*). More positive values of this contrast indicate a greater sequential congruency effect. In the current Simulation, (only) the stimulus determines whether to boost or not; block would make no sense in this case as there is just a single block type (50% congruent stimuli).

## Results and discussion

**Figure 5:**
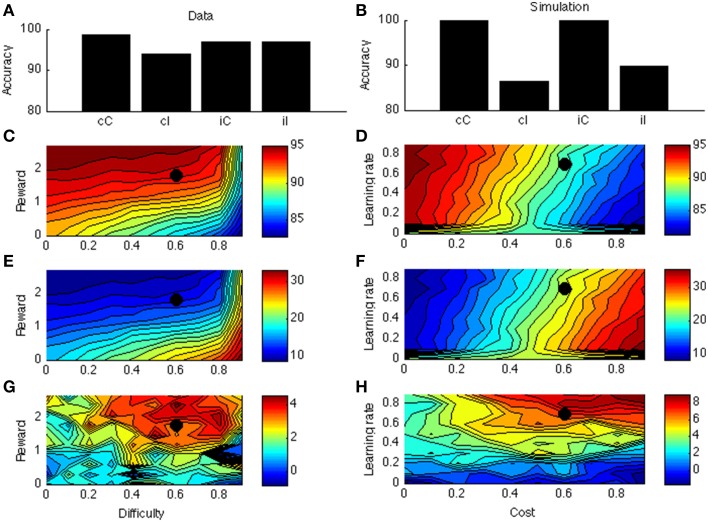
**(A)** Sequential congruency effect from Fischer et al. (2008). On the X-axis, small letters (c, i) indicate previous-trial congruency; capital letters (C, I) indicate current-trial congruency. **(B)** Simulated sequential congruency effect. **(C)** Simulated accuracy across different levels of reward and task difficulty. **(D)** Simulated accuracy across different levels of learning rate and cost. **(E)** Simulated congruency effect across reward and task difficulty. **(F)** Simulated congruency effect across learning rate and cost. **(G)** Simulated sequential congruency effect across reward and task difficulty. **(H)** Simulated sequential congruency effect across learning rate and cost. Black dots in **(C–H)** indicate the parameter used to generate **(B)**.

Figure 5A displays accuracy as a function of current and previous congruency from a typical data set (Fischer et al., 2008). There is a congruency effect and an interaction with previous-trial congruency (sequential congruency effect). The same is true in the model (Figure 5B).

Figures 5C–F are similar to Figures 4C–F. Figure 5G displays the effect of reward and task difficulty on the sequential congruency effect. The sequential congruency effect is only present if both reward and difficulty are sufficiently high. Only in this case does the model consider it worth “boosting,” or changing its strategy depending on the previous-trial congruency. Figure 5H shows the effect of learning rate and cost. In the blockwise PC effect, the influence of learning rate was small, because the effect builds up across an entire block; the effect disappeared only for very low learning rates (see Figure 4H). This is not the case for the sequential congruency effect (Figure 5H); because the effect depends on the previous trial only (even if averaged across an entire block), it is only present when learning is rapid.

In the model the blockwise PC and sequential congruency effect both derive from local estimation of effort requirements. Another way of seeing this is that Equation (6) takes an exponentially weighted average of the net value (reward— effort cost) of boosting across all earlier trials, with largest weight on the last trial. In a way, the sequential congruency effect is a local (one trial) blockwise effect. However, they are different effects: The learning environment for the sequential congruency effect is just a single trial (i.e., the previous one), making it much less robust than the PC effect (compare noise in Figures 5G, 4G; and the large effect of learning rate in 5 h but not in 4 h). In this way, dissociations between the two effects, where the PC extends across tasks but the sequential congruency effect does not (Funes et al., 2010), can be accounted for. Another interesting distinction between the two effects relates to the required interval [e.g., measured as response-stimulus interval (RSI)] between trials to obtain the effect. As the sequential congruency effect depends on rapid learning between trials, it may not (robustly) occur for very short RSIs, for which there is indeed some evidence (Notebaert et al., 2006). In contrast, the proportion congruency effect should not (or much less so) depend on RSI. This dissociation remains to be tested explicitly.

## Simulation 4: effort discounting

### Methods

Recent studies on effort discounting typically use a T-maze setup (Salamone et al., 1994; Denk et al., 2005) where animals choose between a low-reward (LR) food and a high-reward (HR) food. Each option is located in one arm of the T-maze. The HR choice is also more difficult because obtaining the food requires climbing over a barrier. Control animals go for the HR choice, but DA-impaired animals instead choose the LR; hence, preference reverses as a result of DA impairment. This setup is simulated here. We consider the effect of focal (in VS, e.g., with 6-OHDA) or systemic blocking of dopamine (e.g., with haloperidol) (Salamone et al., 1994). Both focal and systemic lesions block dopaminergic input into VS and thus impair the limbic loop (Salamone and Correa, 2012). Importantly, choice is not influenced by the dopaminergic manipulation when there is no barrier; in this case, animals choose the HR. Hence, the effect of dopaminergic impairment is not due to learning, memory, attentional, or motor-related factors. Also, the effects of VS lesion are very different from the effects of food devaluation (e.g., prefeeding, Salamone and Correa, 2012). The animals just don't want to climb over the barrier to obtain the food anymore after dopaminergic (VS) lesion.

We first trained the model to make it appreciate the difference between the HR and LR choices. On half of the training trials, the model is shown the left (but not the right) arm of the maze, with the choice for a left response, right response, or stay response (choosing not to enter any of the two arms); see Figure 3C. In these trials, the animal must choose between going left and staying where it is, perhaps because climbing is too costly. On the other half of the training trials, it is shown the right arm of the maze (but not the left); in these trials, the animal chooses between going right and staying where it is. Thus, we mimicked the training procedure where animals are exposed to the two arms separately to estimate each arm's value.

We arbitrarily designated the left arm in the maze as the LR and the right arm as the HR choice. When there was a barrier, the HR choice was also more difficult to obtain. In particular, the implemented S-R matrix was then

$$w^{\text{SR}}=\left(\begin{array}{ccc} 10 & 0.01 & 0\\ 0 & 0.8 & 1 \end{array}\right)$$

with rows 1 and 2 corresponding to left (LR) and right (HR) stimulus respectively; and columns 1 and 3 to left and right action, respectively. The middle column corresponds to remaining in the middle between the two arms, the “staying where it is” option. In this way, the Go left action will typically lead to reaching the left arm, but a Go right action will require boosting to overcome ending up in the middle.

To model the situation without a barrier, the S-R matrix is symmetric for Go left and Go right actions:

(9) $$w^{\text{SR}}=\left(\begin{array}{ccc} 10 & 0.01 & 0\\ 0 & 0.01 & 10 \end{array}\right)$$

Simulation 4 implements a choice paradigm in which an animal is queried which of two choices (LR or HR) it prefers. For this purpose, we also calculate values of situations *s* (*V* unit in Figure 2) on trial *n*:

$$V_{n}^{\pi }(s)\;=V_{n-1}^{\pi }(s)\;+\;\alpha \;\left(R-c\cdot x_{Cost}-V_{n-1}^{\pi }(s)\right)$$

Training consisted of 200 trials. After training, we calculate preferences for the two options (LR or HR) from their respective values *V*. We again used a softmax for this choice:

(10) $$\mathrm{Pr}(HR)=\frac{\exp\left(\gamma V_{200}^{\pi }(HR)\right)}{\exp\left(\gamma V_{200}^{\pi }(HR)\right)+\exp\left(\gamma V_{200}^{\pi }(LR)\right)}$$

For comparison with empirical data in which control animals were compared with dopamine-impaired animals (6-OHDA lesion in VS) (Salamone et al., 1994), we implemented a control model (as in Simulations 1–3), and a model with a dopamine-depleted limbic loop. In this case also, and consistent with experimental rodent and human paradigms (Walton et al., 2002; Shiner et al., 2012), animals were trained with an intact system. To mimic the impaired gate of VTA to limbic loop leading to loss of effort, values (in Equation 10) were now considered over *Q*(s, No Boost) values rather than across *V*(*s*) values. The rationale is that the animal must estimate the values of left and right arm choices. These values are *V*(*s*) = Pr(Boost | *s*) ^*^ *Q*(*s*, Boost) + Pr(No Boost | *s*) ^*^ *Q*(*s*, No Boost), which simply becomes *Q*(*s*, No Boost) if Boosting is unavailable. These values can be obtained either with a model-free procedure (Monte-Carlo sampling of the world's contingencies) or a model-based procedure (Daw et al., 2005; Solway and Botvinick, 2012). In the latter, the animal starts from the *Q*-values and simulates possible consequences to obtain the *V*-values. To approximate these two possibilities in a simple way, choosing in the intact model occurred based on the *V*(*s*) values; in the dopaminergically lesioned model, it occurred based on the *Q*(*s*, No Boost) values.

Average performance is calculated across 100 model replications. To explore the model's parameter space, we again crossed reward and task difficulty, and learning rate with cost. The same parameter settings were used as in Simulations 2 and 3.

## Results and discussion

**Figure 6:**
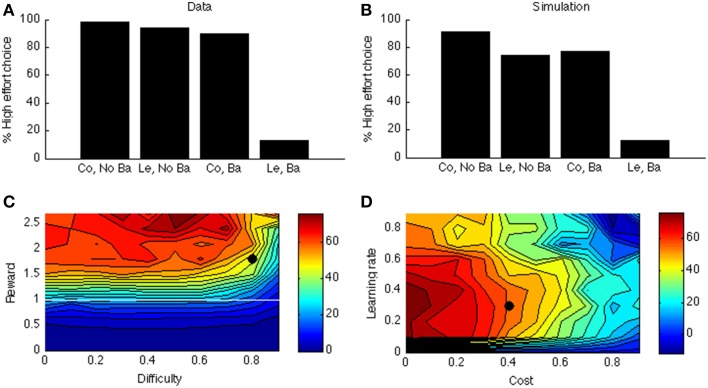
**(A)** Data from Salamone et al. (1994). On the X-axis, Co, Control; Ba, Barrier; Le, Lesion. On the Y-axis is the percentage of high-effort (rather than low-effort) choices. **(B)** simulated interaction for one parameter point (indicated by a black dot in **C,D**). **(C)** Simulated interaction across different levels of reward and difficulty. **(D)** Simulated interaction across different levels of learning rate and cost.

Figure 6A shows the data of a typical experiment (Experiment 2, test week 1 from Salamone et al., 1994). When there is no barrier, both control and lesioned animals go for the HR arm (first two bars); when there is a barrier (last two bars), the lesioned animals avoid the HR arm. Figure 6B shows the corresponding simulated two-way interaction. The first two bars simulate the no-barrier case (symmetric weight matrix, Equation 9). As in the empirical data, there is a clear preference for the HR arm. In contrast, when a barrier is introduced (last two bars), the control model still prefers the HR arm, but the lesioned model avoids it.

As before, to explore the parameter space, we vary the reward of the difficult option. In Simulation 4, we call this the HR choice, but obviously when reward <1 (white line in Figure 6C) it is no longer the high reward choice (because it is then lower than for the LR choice). However, for consistency we maintain the terminology. The clearest effect is that of reward. If the HR is lower than the LR, the interaction is not present (below white line in Figure 6C). It is indeed adaptive not to choose the “high reward” option in this case.

Figure 6D shows how this interaction depends on learning rate and cost. Like before, when learning rate is zero, there is no effect at all (model cannot learn to act adaptively). Finally, there is a clear effect of cost: When the cost of boosting becomes too high, the model does not want to work anymore. At first glance, it seems odd that a mere change in gain can cause a preference reversal. However, it can be explained by the following analogy: Suppose one can choose between a task with success probability 0.9 and reward of 1 when successful, and a task with success probability 0.5 and reward of 1.5 for success. In this case, the expected value of the first task is higher. Suppose we now increase the gain of both tasks, so their success probabilities become much higher, say 0.99 and 0.9, respectively. Then, the expected value of the second task is higher, which leads to a preference reversal. A similar scenario occurs in our simulation in which DA impairment decreases the probability of success of the difficult task.

## Simulation 5: balancing rewards and costs in variable-interval schedules

### Methods

The next physical effort paradigm we simulate is responding in behavioral reinforcement schedules. A classic finding is *hyperbolic responding*: Response rate increases rapidly as a function of average reward rate at lower levels of reinforcement, but levels off at higher reinforcement rates (Catania and Reynolds, 1968; Harris and Carpenter, 2011). An interpretation is that responding faster is well worth it at lower levels of responding, but as the cost of responding increases, equilibrium is obtained, balancing reward and effort. Response rate does not increase beyond that point. This behavior can be described by a hyperbolic curve *z* = *a*_1_*x*/(1+*a*_2_*x*) with *x* representing reinforcement rate and z representing response rate.

Following modeling work of Niv et al. (2007) and empirical work of Catania and Reynolds (1968), we implement a variable-interval reinforcement schedule. At each time step (out of 100), there is a probability *p* of reinforcement (when a response is given). Hence, the average delay between reinforcements is 1/*p*, and so *p* (actually, 1/*p*) is our manipulation of delay. The probability *p* varies from 0 to 1 in steps of 0.02 across simulations. To implement a cost associated with fast lever pressing, we used a simplified version of a scheme implemented in earlier modeling work (Niv et al., 2007), with cost equal to a constant (systematically manipulated from 0 to 9) for the minimum delay (of 1) between lever presses (simulated responses) and cost equal to zero otherwise. Similar results were obtained with smoothed versions of this cost function.

The response mapping is shown in Figure 3D. Delay between lever presses is implemented by a tapped delay line (Brown et al., 1999). *Q*-values *Q*(delay, Boost) and *Q*(delay, No Boost) are calculated for the Boost and No Boost options, respectively. Ten time points are implemented (so delay varies between 1 and 10). Parameter settings were the same as in earlier simulations. The same equations and formalism as before were used, but the weight matrix w^SR^ consists of a vector of 1's, so responding (lever pressing) takes a particularly simple form:

$$\mathrm{Pr}(lever\;press)=\frac{\exp\left(ACC_{boost}+bias\right)}{1+\exp\left(ACC_{boost}+bias\right)}$$

with bias = −3, implementing a tendency to withhold responding except when boosted by the limbic loop. Parameters were the same as before except that γ = 6. Hundred simulations are implemented for each level of *p*, with 200 trials each. Responses are recorded only in the last 50 trials, when learning is stabilized.

As one measure of model performance, we consider hyperbolic goodness-of-fit (*R*^2^ value). If the probability of responding increases linearly, this results in a low fit of the hyperbolic model; indeed, the hyperbolic model cannot mimic the linear model because it has no intercept term. Hence, this *R*^2^ value summarizes the extent to which response rate “flattens off” (hyperbolically) rather than increases linearly with increasing reinforcement rate.

As a more direct measure of model performance, we consider the number of alternations between responding and not responding the model makes. Each run (i.e., bout of uninterrupted responses) is ended by such an alternation, so the shorter the runs, the more alternations there will be. The cost function dictates that every boost (and therefore every response) immediately after a boost is costly but otherwise, it is not. A model that takes this information appropriately into account always alternates responding with not responding, leading to 50 runs (because 50 trials are recorded). A model that responds on every trial produces just one run. Hence, a higher number of runs means more sensitivity to the cost function. For this dependent variable, we produce heat maps like in Simulations 2–4, now systematically exploring reward and cost parameters. Task difficulty has no meaning in these simulations, so the parameter space exploration process is reduced.

## Results and discussion

**Figure 7:**
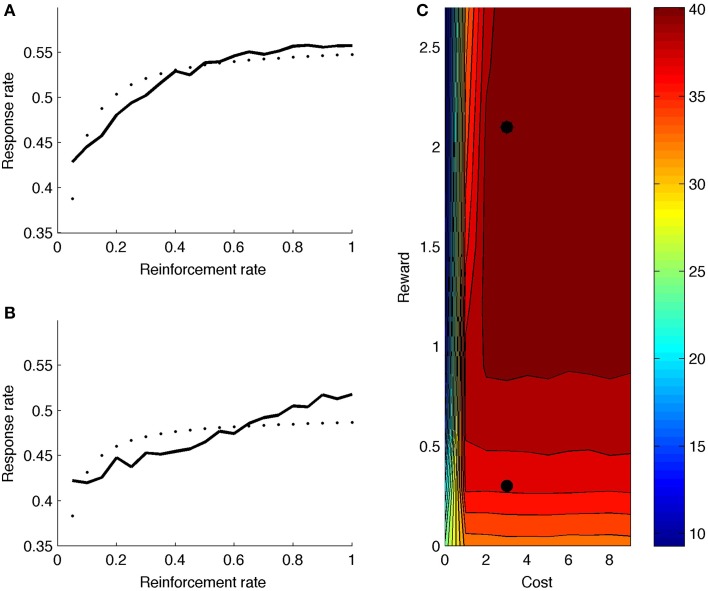
**(A)** Response rate curve with reward rate = 2.1 (corresponding to upper black dot in **C**). **(B)** Response rate curve with reward rate = 0.3 (corresponding to lower black dot in **C**). **(C)** Mean number of runs for different reward and cost values. Dotted line is best-fitting hyperbolic curve.

Figure 7A shows model response rate as a function of reward rate (full black line; reward and cost parameter indicated by upper black dot in heat map of Figure 7C). The fitted hyperbolic curve is illustrated with small black dots in 7a. The hyperbolic goodness-of-fit equals *R*^2^ = 0.72. The model learns that it should not boost on the very first time step after reinforcement, but boosting is valuable right after, especially for high reward rates. The net effect is a negatively accelerated response curve: Responding increases quickly for low response rates (X-axis in Figure 7A), but levels off and eventually balances reward rate with effort. Figure 7C (upper black dot) shows the mean number of runs for this parameter point, averaged across reward rate values (X-axis in Figure 7A). The model generates a high number of runs, almost alternating responding with not responding.

Figure 7B shows the response rate curve for a lower reward value (lower black dot in heat map of Figure 7C). The hyperbolic goodness-of-fit is lower in this case (*R*^2^ = 0.45). Also, the number of runs is much lower (Figure 7C); the model does not alternate responding and not responding.

Turning to the complete heat map, the model generally produces more runs for higher rewards, because boosting is more valuable when reward is higher. Further, if cost is very low, the model doesn't alternate: If there is no cost associated to boosting, one can respond more often, producing fewer runs. Again, the model combines reward and cost information to determine its optimal strategy.

In addition to an opportunity (or time) cost, as implemented here, various other costs may be at work, including a switch cost as implemented by Niv et al. (2007). In the current paradigm, this is not possible, but the setup can easily be extended such that it does (i.e., when multiple responses are available). Future work can explore the interactions between the different types of cost.

## General discussion

We modeled effort exertion both from an optimality and from a neurocomputational perspective. We demonstrated that adaptive boosting can optimize value across reward, cost, and task difficulty. A major debate on the functionality of the limbic loop and its components concerns whether it codes for value or effort (Knutson et al., 2005; Croxson et al., 2009; Bissonette et al., 2013). There is overwhelming evidence for both (Botvinick et al., 2009; Croxson et al., 2009; Bissonette et al., 2013; Kurniawan et al., 2013), suggesting that an integrative view is needed. The current work provides one, and shows that value and effort can be integrated into a single RL framework. As Holroyd and Yeung (2012) noted “the proposal that ACC motivates goal-directed behavior relies on intuitive but computationally imprecise terms such as ‘effort’ and ‘energy’ (p. 122). The current model deals with this problem by providing a plausible computational mechanism for adaptive effort exertion.

The model contained a number of simplifications, or formulated more productively, avenues for future extension. In the neurocomputational model, boosting is binary rather than continuous. Relatedly, we adopted a tabular RL approach, where a single stimulus or state updates its value on any trial. Both can be overcome by combining RL with function approximation methods (Sutton and Barto, 1998). Second, we implemented effort by gain modulation. Other options include choosing between model-based and model-free processing (Daw et al., 2005), tolerating punishment in the face of upcoming rewards (Tanaka et al., 2004), or increasing cortical learning rate (Behrens et al., 2007; Silvetti et al., 2013a). All of these are reasonable options, depending on task specifics. They can be considered as meta-options in the terminology of the current paper: In a hierarchical reinforcement learning framework, the type of control to be exerted can be considered a higher-level choice to be made (Shenhav et al., 2013; Holroyd and McClure, 2015). Third, we have not exhaustively mapped the effort network (e.g., basolateral amygdala; Floresco and Ghods-Sharifi, 2007). Fourth, the model estimates just a single characteristic of the reward distribution, namely the reward mean. However, also other distributional characteristics, most prominently reward variance (i.e., risk) have received strong attention in recent years (Preuschoff et al., 2006). Reward variance has been proposed as a key determinant for choosing between behavioral controllers (Daw et al., 2005), and may be important in choosing the amount of effort to put into a task. One final limitation is that, because of our “breadth first” approach, we were not able to distinguish between variants of the specific tasks and experimental paradigms that we modeled. For example, we could not here address differences between Stroop and Simon tasks (in the cognitive domain), nor make detailed comparisons of ACC vs. VS lesions (Salamone et al., 1994; Walton et al., 2002). Such issues will be addressed in future work.

### Value

A large literature associates ACC with performance monitoring (Botvinick et al., 2001; Holroyd and Coles, 2002; Ridderinkhof et al., 2004; Verguts and Notebaert, 2009). Recent models propose that a core function of ACC is value estimation, or that ACC is a *Critic* (Alexander and Brown, 2011; Silvetti et al., 2011, 2014). These models account for several performance monitoring findings in ACC, including error detection (Falkenstein et al., 1991; Gehring et al., 1993), error likelihood (Brown and Braver, 2005), and conflict (Botvinick et al., 2001). However, such models consider the reward only of external stimuli, and thus cannot account for the basic effort-related findings discussed in this paper. For example, this value perspective predicts more ACC activation for cues indicating easy rather than difficult stimuli (because subjects prefer easy stimuli; Vassena et al., 2014). In contrast, the current model considers a different type of value, the (*Q*-)value of boosting, which is consistent with more activation for cues predicting difficult stimuli (cf. Figure 1), as empirically observed (Vassena et al., 2014).

### Effort

Recent work already investigated effort processing from an RL perspective (Niv et al., 2007; Rigoux and Guigon, 2012; Shenhav et al., 2013; Holroyd and McClure, 2015). Rigoux and Guigon (2012) implement a model that optimizes a combination of reward and effort from an engineering control theory perspective. However, they did not conceptualize modulating an action (such as boosting in the current model) as a separate process that is subject to RL. Moreover, they did not specify a neural substrate for their model. Niv and co-workers also investigated effort from an RL perspective (Niv et al., 2007). Like in the current model, they conceptualized effort investment as an option subject to RL principles. In particular, they considered the delay between actions (e.g., lever presses in a variable-interval schedule) as a choice with two types of (effort) cost attached to it (opportunity cost, energetic cost). A system of Bellman equations was solved to find the optimal delay. It is not clear, however, how animals can choose an abstract quantity like delay, or optimize the corresponding equations. In our model, delay is instead determined implicitly, in the sense that different delays (different time intervals since the last lever press) have a different *Q*-value. Furthermore, in our model, these values are computed online using locally available information. Finally, the Niv et al. (2007) model focused on physical effort only. Another recent model focusing on physical effort is by Holroyd and McClure (2015). In their model, ACC exerts hierarchical control over striatum, and (rodent) prelimbic cortex exerts control over ACC. Control is here implemented by ACC suppressing effort costs in striatum if doing so is worthwhile for obtaining higher rewards.

It was recently proposed that ACC calculates the expected value of control (Shenhav et al., 2013). In particular, ACC calculates the value of each possible control signal and its intensity. However, the signals and their intensities remained largely unspecified, making it difficult to determine how, after choosing a signal and corresponding intensity, this signal and intensity are implemented and used by the cognitive system. Also, this paper focused on cognitive effort only. Instead, our model is the first to simulate neural and behavioral data in a broad range of species and tasks. This broadness derives from our modular approach, where task and effort investment are two separate learning problems, each with its own utility function.

Despite several differences, a common theme running through many of these proposals (Niv et al., 2007; Shenhav et al., 2013; Holroyd and McClure, 2015), including the current one, is that effort investment is considered as an action (called option in this paper), that is subject to RL principles, just like basic actions are. Another commonality is the notion of hierarchy (Shenhav et al., 2013; Holroyd and McClure, 2015). Also in our model, processing can be considered to be hierarchical in the sense that effort consists of optimizing the (gain) parameters in the (cortical) task network. Future empirical work should disentangle the various options that have been proposed.

### Cognitive control

In the model, only the value of current stimulus and option are updated. For this reason, it can be formulated as Hebbian learning. In this sense, it is similar to our earlier model (Verguts and Notebaert, 2008), which formulated cognitive control as Hebbian learning modulated by evaluative signals (e.g., reward or conflict). However, the current model is much broader, covering not only cognitive but also physical effort. Second, the current model shows that reinforcement-modulated Hebbian learning not merely fits the data, but also provides a perspective on *why* such learning is appropriate. In this sense, it provides both a descriptive and normative perspective on effort allocation and control.

### Limbic, motor, and cognitive loops through BG

Besides limbic and motor loop, a third well-described loop is the loop via the dorsolateral prefrontal cortex and head of the caudate nucleus (Alexander et al., 1986; Pierce and Kumaresan, 2006). This loop also provides a modulatory influence on cortex, for example, by gating information and thus enabling working memory (O'Reilly and Frank, 2006). We propose that the dopaminergic pathway itself modulates the modulatory BG loops, thus allowing the BG loops to learn. However, dopamine has a second role in supporting performance itself via a different pathway (Braver and Cohen, 2000). Both roles have been documented (Schultz et al., 1997; Salamone and Correa, 2012; Shiner et al., 2012). Further, a gradient exists in the SN/VTA complex, with more ventromedial dopamine neurons responding to reward-related events, and more dorsolateral neurons responding to task-relevant events (Matsumoto and Takada, 2013).

### Noradrenaline and the signal-to-noise ratio

Our model implemented effort by adaptively changing the SNR of the stimulus-to-action mapping. Modulation by changing the SNR was also proposed in adaptive gain theory (Aston-Jones and Cohen, 2005). This model proposed that noradrenaline increases SNR when a task-relevant stimulus is presented (Usher et al., 1999). Related models proposed that noradrenaline adapts SNR based on reward and response conflict to balance exploration vs. exploitation (McClure et al., 2005; Cohen et al., 2007). However, even though adaptation was dynamic in these models, it was hard-wired under what conditions SNR changes are needed. If noradrenaline is to change SNR, it needs similar adaptive learning modulation as described in the current paper, perhaps delivered by dopamine as well.

## Conclusion

Our model integrates a number of factors that have usually been treated independently: benefit (reward) and cost (effort) computation; cognitive and physical effort; dopamine and its dual role in cognition; the modulatory role of the BG loops; motivational and value processing in limbic structures (ACC, VS); and monitoring and action functions of ACC. This allows for theoretical clarity and unification and leads to several empirical predictions. Many were already mentioned throughout the text; we here enumerate a few broader predictions. First, investing effort is both learnable and context-dependent. The cognitive control literature has generated a huge number of context-dependent cognitive control effects (Crump et al., 2006; Blais et al., 2007; Notebaert and Verguts, 2008; Braem et al., 2011). We predict more generally that context-specificity is a core feature of effort investment. Another prediction is that exerting cognitive and physical effort relies on a similar circuitry. Hence, impairments in cognitive and physical effort exertion would be correlated. Another prediction is that connectivity between stimulus and action processing areas increases when the subject is cued that a difficult task (requiring high effort) is coming up. Furthermore, this connectivity should be modulated via limbic loop activation. This may be tested by fMRI functional connectivity methods. Finally, the model connects to clinical syndromes. For example, ADHD is characterized by blunted effort exertion. Earlier models of ADHD focused on reward learning (Williams and Dayan, 2005; Cockburn and Holroyd, 2010; Silvetti et al., 2013b) or motivation (Sonuga-Barke et al., 2010). In the current model, reward learning and motivation are related but separate components; even though they are behaviorally correlated, different neural pathologies can be characterized by different performance deficiencies. Similarly, because of the modularity of effort, it should in principle be possible to have impaired effort processing (Treadway et al., 2009) but intact delay processing; this also remains to be tested. To sum up, the model provides a point of departure for bridging brain and behavior in the ill-understood but ubiquitous production of cognitive and physical effort, across species, in health and disease.

### Conflict of interest statement

The authors declare that the research was conducted in the absence of any commercial or financial relationships that could be construed as a potential conflict of interest.

## Appendix

Suppose task-relevant input (e.g., ink color in the Stroop task) has a bottom-up influence of strength 1 to the response level; and task-irrelevant input (e.g., words in the Stroop task) has a bottom-up influence of *I* (typically *I* > 1). To counteract the influence of the irrelevant dimension, top-down signals bias the task-relevant input with a gating strength of *Z* and 1-*Z* for task-relevant and irrelevant dimensions, respectively (Botvinick et al., 2001). Consequently, when a congruent stimulus is presented, the total input to the correct and incorrect response are, *Z*+(1-*Z*)*I* and 0, respectively; and when an incongruent stimulus is presented, total input to the correct and incorrect response are *Z* and (1–*Z*)*I*, respectively. This can be implemented with the S-R matrix

$$\left(\begin{array}{cc} Z+(1-Z)I & 0\\ (1-Z)I & Z \end{array}\right)$$

with rows 1 and 2 for congruent and incongruent stimuli, respectively, requiring response (column) 1 and 2, respectively (as in the main text). This matrix is consistent with Figure 3B and is approximated by the one we used in Simulations 2 and 3.
